# The frequency of the predominant Jewish mutations in *BRCA1* and *BRCA2* in unselected Ashkenazi colorectal cancer patients

**DOI:** 10.1054/bjoc.2000.1598

**Published:** 2001-02

**Authors:** R Chen-Shtoyerman, A Figer, H H Fidder, P Rath, L Yeremin, S Bar Meir, E Friedman, L Theodor

**Affiliations:** Gastroentrology, Chaim Sheba Medical Center, Tel-Hashomer, 52621, Israel; The Oncology Department, Rabin Medical Center, Petach Tiqvah, Israel; Oncology Institutes, Israel; Susanne Levy Gertner Oncogenetics Unit, Chaim Sheba Medical Center, Israel

**Keywords:** Ashkenazi Jews, colorectal cancer, *BRCA1*, *BRCA2*, 185delAG, 5382insC, 617delT, inheritedredisposition

## Abstract

It is presently unclear whether carriers of *BRCA1* mutations have an increased risk for colorectal cancer (CRC). To gain insight into this issue, 225 unselected Ashkenazi Jewish CRC patients were tested for the presence of the three common Jewish *BRCA1/2* germline mutations: 185delAG and 5382insC (*BRCA1*) and 6174delT (*BRCA2*). A total of four carriers was found (4/225, 1.78%). This frequency is similar to the estimated normal Ashkenazi population frequency, thus suggesting that these specific mutations do not contribute to CRC predisposition.© 2001 Cancer Research Campaign http://www.bjcancer.com


*British Journal of Cancer* (2001) **84**(4), 475–477
© 2001 Cancer Research Campaign
doi: 10.1054/ bjoc.2000.1598, available online at http://www.idealibrary.com on IDEAL®

http://www.bjcancer.com

Short Communication

### The frequency of the predominant Jewish mutations in *BRCA1* and *BRCA2* in unselected Ashkenazi colorectal cancer patients

**R Chen-Shtoyerman[1], A Figer[2], HH Fidder[1], P Rath[3], L Yeremin[2,4], S Bar Meir[1], E Friedman[4] and L Theodor[1]**

Gastroenterology[1] and Oncology Institutes[3] and the Susanne Levy Gertner Oncogenetics Unit[4], Chaim Sheba Medical Center, Tel-Hashomer, 52621; The Oncology Department, Rabin Medical Center, Petach Tiqvah[2], Israel

**Summary** It is presently unclear whether carriers of *BRCA1* mutations have an increased risk for colorectal cancer (CRC). To gain insight into this issue, 225 unselected Ashkenazi Jewish CRC patients were tested for the presence of the three common Jewish *BRCA1/2* germline mutations: 185delAG and 5382insC (*BRCA1*) and 6174delT (*BRCA2*). A total of four carriers was found (4/225, 1.78%). This frequency is similar to the estimated normal Ashkenazi population frequency, thus suggesting that these specific mutations do not contribute to CRC predisposition. © 2001 Cancer Research Campaign http://www.bjcancer.com

**Keywords**: Ashkenazi Jews; colorectal cancer, *BRCA1*; *BRCA2*; 185delAG; 5382insC; 617delT; inherited predisposition

The co-occurrence of breast cancer (BC) and colorectal cancer (CRC) has previously been documented (Phipps and Perry, 1989; Ford et al, 1994; Schoen et al, 1994; Slattery and Kerber, 1994; Olsen et al, 1999). Women with a history of BC were found to have an increased risk for developing subsequent CRC (Rozen et al, 1986; Schoen et al, 1994). Such an association between BC and CRC could arise due to a genetic predisposition (Slattery and Kerber, 1994; Stoll, 1998). Several lines of evidence point to a possible contribution of mutations within the inherited BC susceptibility gene, *BRCA1*, to CRC pathogenesis: allelic losses at the *BRCA1* locus, putatively targeting this tumour suppressor gene, have been detected in almost 50% of sporadic CRCs (Garcia-Patino et al, 1998); Individuals within *BRCA1*-linked families have an increased risk for developing CRC – the relative risk of *BRCA1* mutation carriers (by haplotype analysis) for CRC was found to be 4.11 (Ford et al, 1994) and the risk for developing CRC in relatives of familial BC patients is increased over that of the general population (Phipps and Perry, 1989; Slattery and Kerber, 1994; Burke et al, 1997; Olsen et al, 1999). However, the increased risk for developing CRC in patients with familial BC is not uniformly reported by all investigators (Anderson and Badzioch, 1993; Lin et al, 1999). A high carrier rate of *BRCA1/2* families in a defined population permits a comparison of mutation frequencies between affected CRC individuals and the general population.

Among Ashkenazi (East European) Jews, three mutations in the *BRCA1* and *BRCA2* genes, account for the majority of inherited BC predisposition: 185delAG and 5382insC (*BRCA1*) and 6174delT (*BRCA2*). Furthermore, these mutations occur at a rate of about 2.5% among the general Jewish Ashkenazi population (Struewing et al, 1995, 1997; Roa et al, 1996; Fodor et al, 1998). These facts and the lack of conclusive evidence for an increased CRC risk in BC families, prompted us to directly analyse the relative contribution of these mutations to the pathogenesis of CRC in Ashkenazi Jews.

*Received 22 March 2000*
*Revised 18 October 2000*
*Accepted 27 October 2000*

*Correspondence to:* L Theodor

### MATERIALS AND METHODS

### Subjects

225 consecutive Ashkenazi CRC patients were included in the study: 125 men and 100 women. The patients were diagnosed at the Sheba and Rabin Medical Centers and had pathologically confirmed tumours. The Ashkenazi descent was ascertained at least three generations back. The mean age at diagnosis was 65.3 ± 17.2 years, and the mean age at the time of study was 73.5 ± 11.0 years.

The study was approved by the Institutional Review Board at both medical centres. All participants signed a written informed consent, and a detailed questionnaire, with special emphasis on cancer family history was filled out.

### Molecular analysis

DNA was extracted from peripheral blood leukocytes using standard techniques and analysis for the three predominant mutations was performed using PCR and restriction fragment length polymorphism, as previously described (Rohlfs et al, 1997) and adopted by us (Bruchim Bar-Sade et al, 1998).

### Statistical analysis

Carrier frequency rates were compared between the CRC study group and published data regarding the general Ashkenazi population, using Fisher's Exact Test.

### RESULTS

Overall, 4 out of the 225 patients tested (1.78%) were *BRCA1/BRCA2* mutation carriers. The carriers consisted of two

**Table 1** Clinopathological data of *BRCA1/2* mutation carriers

| Patient | Sex | BC personal diagnosis age | BC diagnosis age in 1st degree relative | CRC diagnosis age | *BRCA1/2* mutation status |
|---|---|---|---|---|---|
| C1 | F | 49 | – | 67 | 6174delT |
| C2 | F | 62 | – | 85 | 6174delT |
| C3 | M | – | 32 | 72 | 185delAG |
| C4[a] | M | – | – | 75 | 5382insC |

[a]Patient C4's mother was diagnosed with endometrial cancer, diagnosis age unknown.

**Table 2** Carrier frequency of the three predominant Jewish *BRCA1/2* mutations

| Mutation | CRC patients | Healthy controls (Struewing et al, 1997) | *P* value[a] |
|---|---|---|---|
| 185delAG | 1/225 (0.44%) | 41/5318 (0.77%) | NS |
| 5382insC | 1/225 (0.44%) | 20/5318 (0.38%) | NS |
| 6174delT | 2/225 (0.88%) | 59/5318 (1.11%) | NS |
| Total | 4/225 (1.78%) | 120/5318 (2.26%) | NS |

[a]Statistical analysis using Fisher's Exact Test.

males and two females. One male patient was a 185delAG *BRCA1* mutation carrier (0.44%) and the other male a 5382insC *BRCA1* mutation carrier (0.44%). The two female patients (0.88%) harboured the 6174delT *BRCA2* mutation. Of note, 3 out of the 4 mutation carriers had either a personal or family history of BC. The relevant clinical data of these carrier individuals are shown in Table 1. The differences between the mutation carrier frequencies in this patient population were not statistically significant compared with those of the general population (Struewing et al, 1997), as calculated using Fisher's Exact Test (Table 2).

Among the remaining 98 non-carrier females with CRC, only one patient (1.02%) had a primary diagnosis of BC at age 52 years and a diagnosis of CRC 18 years later. 13 out of the 131 (9.92%) CRC patients, who completed the family history questionnaire, reported BC in a first degree family member. This rate is in concordance with the rates of BC in the general Jewish population (Bar-Chana et al, 1996).

### DISCUSSION

Previous analysis of the three common Jewish *BRCA1/2* germline mutations in a large, unselected group of Jewish Asheknazi individuals did not find an increased risk for developing CRC among mutation carriers (Struewing et al, 1997). Lin et al reported that the lifetime risk for CRC in 32 American *BRCA1/2* families was similar to the risk in the general population (1999). Our results support these data by analysis of unselected CRC patients, and complement previous studies performed on individuals from high-risk families. Of note, had we excluded patients with personal or familial history of BC from the patient cohort as well as from the control group analysed, the lack of association between these mutations and CRC would be even more striking. Taken together with the recent publications showing these specific mutations do not increase the risk for prostate cancer in this ethnic group (Lehrer et al, 1998; Hubert et al, 1999; Vazina et al, 2000), we can conclude that the predominant Ashkenazi Jewish *BRCA1* and *BRCA2* mutations do not contribute to the pathogenesis of CRC. Thus, it seems that the two major indications for performing *BRCA1/2* genetic testing in men are the personal risk for developing BC (Struewing et al, 1999) and the risk of transmitting the mutated alleles to their daughters.

### ACKNOWLEDGEMENTS

This work was performed in partial fulfillment of the requirements for the Ph.D. degree from the Sackler School of Medicine at the Tel-Aviv University for Rakefet Chen-Shtoyerman with the support of the Lizzie and Arthur Lowenstein Doctoral Fellowship in Medicine.

### REFERENCES

Anderson DE and Badzioch MD (1993) Familial breast cancer risks – effects of prostate and other cancers. *Cancer* **72**(1): 114–119

Bar-Chana M, Andreev H and Alon R (1996) *Cancer in Israel*. Israel Cancer Registry 1993, Ministry of Health, State of Israel Publication, Jerusalem

Bruchim Bar-Sade R, Kruglikova A, Modan B, Gak E, Hirsh-Yechezkel G, Theodor L, Novikov I, Gershoni-Baruch R, Risel S, Papa MZ, Ben-Baruch G and Friedman E (1998) The 185delAG *BRCA1* mutation originated before the dispersion of Jews in the Diaspora and not limited to Ashkenazim. *Hum Mol Genet* **7**: 801–806

Burke W, Daly M, Garber J, Botkin J, Ellis Kahn MJ, Lynch P, McTierman A, Offit K, Perlman J, Petersen G, Thomson E and Varricchio C (1997) Recommendations for follow-up care of individuals with an inherited predisposition to cancer. *JAMA* **277**: 997–1003

Fodor FH, Weston A, Bleiweiss IJ, McCurdy LD, Walsh MM, Tartter PI and Browser ST (1998) Frequency and carrier risk associated with common *BRCA1* and *BRCA2* mutations in Ashkenazi Jewish breast cancer. *Am J Hum Genet* **63**: 45–51

Ford D, Easton DF, Bishop DT, Narod SA and Goldgar DE (1994) Risks of cancer in *BRCA1* mutation carriers. Breast Cancer Linkage Consortium. *Lancet* **343** (8899): 692–695

Garcia-Patino E, Gomendio B, Lleonart M, Silva JM, Garcia JM, Provencio M, Cubedo R, Espana P, Ramon y Cajal S and Bonilla F (1998) Loss of heterozygosity in the region including the *BRCA1* gene on 17q in colon cancer. *Cancer Genet Cytogenet* **104**(2): 119–123

Hubert A, Peretz T, Manor O, Kaduri L, Wienberg N, Lerer I, Sagi M and Abeliocvich D (1999) The Jewish Ashkenazi founder mutations in the *BRCA1/BRCA2* genes are not found at an increased rate in Ashkenazi patients with prostate cancer. *Am J Hum Genet* **65**: 921–924

Lehrer S, Fodor F, Stock RG, Stone NN, Eng C, Song HK and McGovern M (1998) Absence of 185delAG mutation of the *BRCA1* gene and 6174delT mutation of the *BRCA2* gene in Ashkenazi Jewish men with prostate cancer. *Br J Cancer* **78**: 771–773

 *© 2001 Cancer Research Campaign*

Lin KM, Ternent CA, Adams DR, Thorson AG, Blatchford GJ, Christensen MA, Watson P and Lynch HT (1999) Colorectal cancer in hereditary breast cancer kindreds. *Dis Colon Rectum* **42**(8): 1041–1045

Olsen JH, Seersholm N, Boice JD Jr, Kruger Kjaer S and Fraumeni JF Jr (1999) Cancer risk in close relatives of women with early-onset breast cancer – a population-based incidence study. *Br J Cancer* **79**(3–4): 673–679

Phipps RF and Perry PM (1989) Familial breast cancer and the association with colonic carcinoma. *Eur J Surg Oncol* **15**(2): 109–111

Roa BB, Boyd AA, Volcick K and Richards CS (1996) Ashkenazi Jewish population frequencies for common mutations in *BRCA1* and *BRCA2*. *Nat Genet* **14**: 185–187

Rohlfs EM, Learning WG, Friedman KJ, Couch FJ, Weber BL and Silverman LM (1997) Direct detection of mutations in breast and ovarian cancer susceptibility gene *BRCA1* by PCR-mediated site-directed mutagenesis. *Clin Chem* **43**: 24–29

Rozen P, Fireman Z and Ron E (1986) Colorectal tumor screening in women with a past history of breast, uterine or obvarian malignancies. *Cancer* **57**(6): 1235–1239

Schoen RE, Weissfeld JL and Kuller LH (1994) Are women with breast, endometrial, or ovarian cancer at increased risk for colorectal cancer? *Am J Gastroenterol* **89**(6): 835–842

Slattery ML and Kerber RA (1994) Family history of cancer and colon cancer risk: the Utah Population Database. *J Natl Cancer Inst* **86**(21): 1618–1626

Stoll BA (1998) Association between breast and colorectal cancers. *Br J Surg* **5**(11): 1468–1472

Streuwing JP, Abeliovich D, Peretz T, Avishai N, Kaback MM, Collins FC and Brody LC (1995) The carrier frequency of the 185delAG mutation is approximately 1 percent in Ashkenazi Jewish individuals. *Nat Genet* **11**: 198–200

Struewing JP, Hartge P, Wacholder S, Baker SM, Berlin M, McAdams M and Timmerman MM et al (1997) The risk of cancer associated with specific mutations of *BRCA1* and *BRCA2* among Ashkenazi Jews. *N Engl J Med* **336**(20): 1401–1408

Struewing JP, Coriaty ZM, Ron E, Liroff A, Konichezky M, Cohen P, Resnick MB, Lifzchiz-Mercerl B, Lew S and Iscorich J (1999) Founder BRCA1/2 mutations among male patients with breast cancer in Israel (letter). *Am J Hum Genet* **65**: 1800–1802

Szabo CI and King MC (1995) Inherited breast and ovarian cancer. *Hum Mol Genet* **4**: 1811–1817

Vazina A, Baniel J, Yaacobi Y, Shtriker A, Engelstein D, Leibovitz I, Zehavi M and Friedman E (2000) The rate of the founder Jewish mutations in *BRCA1* and *BRCA2* in prostate cancer patients in Israel. *Br J Cancer* **83**(4): 463–466

*© 2001 Cancer Research Campaign*

